# Oxidative stability of chelated Sn(II)_(aq)_ at neutral pH: The critical role of NO_3_^−^ ions

**DOI:** 10.1126/sciadv.adq0839

**Published:** 2024-10-02

**Authors:** Shaoyi Zhang, Gokul V. Govindaraju, Chi-Yuan Cheng, Carlo Amorin Daep, Dandan Chen, Cristina Castro, Patrick S. Corrigan, James G. Masters, Long Pan, Guofeng Xu, Tatiana V. Brinzari, Carl P. Myers

**Affiliations:** Colgate-Palmolive, 909 River Road, Piscataway, NJ 08855, USA.

## Abstract

Tin(II) compounds are versatile materials with applications across fields such as catalysis, diagnostic imaging, and therapeutic drugs. However, oxidative stabilization of Sn(II) has remained an unresolved challenge as its reactivity with water and dioxygen results in loss of functionality, limiting technological advancement. Approaches to slow Sn(II) oxidation with chelating ligands or sacrificial electron donors have yielded only moderate improvements. We demonstrate here that the addition of nitrate to pyrophosphate-chelated Sn(II)_(aq)_ suppresses Sn(II) oxidation in water across a broad pH range. Evidence of hydroxyl radical concentration reduction and detection of a radical nitrogen species that only forms in the presence of chelated Sn(II) point to a radical-based reaction mechanism. While this chemistry can be broadly applied, we present that this approach maintains Sn(II)’s antibacterial and anti-inflammatory efficacies as an example of sustained oral chemotherapeutic functionality.

## Teaser

NO_3_^−^ suppresses O_2_/ROS reactivity with chelated Sn(II)_aq_, increasing oxidative stability and sustaining therapeutic efficacy.

## INTRODUCTION

Tin(II) compounds have broad commercial applications, often used as catalysts for small molecule (*1*) and polymer synthesis (*2*, *3*), as oxidative stabilizers (*4*, *5*), as reducing agents for technetium-99m within radiopharmaceutical kits in therapeutic applications (*6*, *7*), or as antibacterial agents in dentifrice formulations (*8*), to name a few. More recently, Sn(II) has been incorporated into water-splitting materials (*9*) and photovoltaics (*10*, *11*), the latter of which is a potential nontoxic replacement for Pb in perovskite solar cells.

However, for Sn(II) to efficiently perform in these applications, the Sn(II) oxidation state must be maintained in both material storage and in use. This challenge is further exacerbated in applications existing in neutral aqueous or O_2_-containing experimental conditions, as Sn(II) readily reacts with H_2_O and O_2_, leading to an oxidized state. The reactive nature of Sn(II) thus represents a substantial developmental limitation in these fields, and a number of recent reports have focused on strategies to limit this process (*9*, *12*–*16*).

**Fig. 1.**
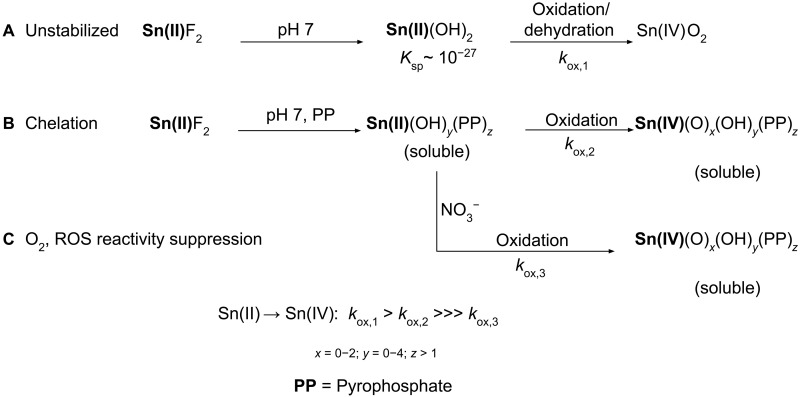
Oxidation and precipitation pathways of Sn(II) in the presence of O_2_ in aqueous solutions. (**A**) Rapid oxidation of unstabilized SnF_2_. (**B**) SnF_2_ chelation with PP slows Sn(II) --> Sn(IV) oxidation. (**C**) NO_3_^−^ further slows chelated-Sn(II) oxidation by suppressing reactivity with O_2_/ROS.

One of the more challenging environments to stabilize Sn(II) is within commercial dentifrice formulations. Since its introduction in the 1950s, stannous fluoride (SnF_2_) has been proven to be clinically safe and effective against caries (*17*–*19*) and gingivitis (*20*), making it one of the most prolific and versatile over-the-counter active ingredients to maintain oral health. It is also the only active ingredient approved by governmental regulatory bodies to treat multiple disease states, which is why research into its oxidative stability is so critical. The challenge of ensuring that SnF_2_ is efficacious is that its individual ions, anticaries F^−^ and antibacterial Sn(II), have pH-driven requirements to be stable and bioavailable. Typical aqueous-based dentifrices are buffered at ~pH 7 to decrease F^−^ adsorption to silica surfaces (*21*–*23*) but, as a result, favor F^−^ over Sn(II). Sn(II) is intrinsically unstable near neutral pH as it readily hydrolyzes, oxidizes, and precipitates, leading to compromised efficacy, as depicted in Fig. 1A (*8*, *24*–*28*). Common approaches to preserving Sn(II) include anhydrous and O_2_-free processing, chelation (Fig. 1B) (*16*, *29*–*31*), sacrificial antioxidants (*32*), or additional Sn(II) salts such as SnCl_2_ (*8*, *33*). All of these methods, however, attempt to slow, block, or compensate for the inevitable reaction of Sn(II) with molecular O_2_ and/or reactive oxygen species (ROS). Expanded drawbacks are listed in table S1. Therefore, an approach that blocks or limits Sn(II)-O_2_/ROS reaction under normal atmospheric conditions at neutral pH would be highly desirable.

Here, we present that the addition of nitrate (NO_3_^−^) to chelated SnF_2_ leads to a profound suppression of Sn(II) oxidation in neutral aqueous solutions (Fig. 1C). The oxidation of Sn(II) and any structural changes were examined through iodometric titrations, ^119^Sn, ^15^N, and ^31^P nuclear magnetic resonance (NMR), and Fourier transform infrared experiments. Sn(II) reactivity with molecular oxygen was evaluated through soluble oxygen and gas-phase O_2_ consumption experiments. Electron paramagnetic resonance (EPR) was used to gain insight into the Sn(II) oxidation mechanism, identifying the presence of both ROS and reactive nitrogen species. Lastly, the antibacterial and anti-inflammatory effects of NO_3_^−^-stabilized chelated Sn(II) solutions were examined to provide foundational evidence to in vivo efficacy. In summary, these data showcase an approach to provide oxidative stability for chelated Sn(II) and demonstrate an example of its sustained functionality.

## RESULTS AND DISCUSSION

### Iodometric titrations

We chose to combine Sn(II) with pyrophosphate (PP) since the resulting complex is soluble at pH 7, provided that the PP:Sn molar ratio is >1.0 (*31*). This allowed for examination of nitrate’s effect on Sn(II) oxidation in partial isolation by minimizing the competing process of precipitation. Note that commercial products containing Sn(II) compounds require demonstrated stability over the full shelf or usage lifetime of a product so as to ensure that material degradation does not affect functionality. Oxidation was then examined by measuring prepared solutions stored at 60°C for 2 weeks to thermally accelerate Sn(II) → Sn(IV) oxidation (*34*), and the resulting [Sn(II)] was quantified through iodometric titrations. The variations and results of these experiments are listed in Table 1. Ligands such as PP are known to slow Sn(II) oxidation by chelation, as is demonstrated by comparing solutions 1 and 2, with 7.5% versus 37% of remaining Sn(II), respectively. When KNO_3_ was included (solution 3), Sn(II) stability improved significantly with 87% stannous recovery after the 2-week time period. Nitrate alone did not provide oxidative stability at pH 7 (solution 4), likely due to dominating hydrolysis reactions. We observed similar trends as solution 3 using NaNO_3_, SnCl_2_, and SnPP (solutions 5, 7, and 8). Further still, if PP was replaced with ethylenediaminetetraacetic acid (EDTA) or trisodium citrate (citrate) (Table 1, solutions 9 to 12), NO_3_^−^ effects were again observed, and Sn(II) recovery increased by ~41 and 49%, respectively. Together, these results indicate that (i) NO_3_^−^ is the primary inhibitor of chelated Sn(II) oxidation, (ii) the effect is not limited to a specific Sn(II) salt, and (iii) the chelating ligand function here is to slow hydrolysis reactions and maintain a soluble Sn(II) complex. Differences in Sn(II) stability among complexes with PP, EDTA, and citrate are attributed to ligand strength and its resulting effect on oxidation protection through chelation, although the action of NO_3_^−^ remains.

**Table 1.** Sn(II) oxidative stability as a function of initial stannous salt, ligand, and additive. Each solution was prepared at pH 7 and stored at 60°C for 2 weeks. Percentage of Sn(II) left in solution was quantified by iodometric titration.

	Solution conditions^*^			
Solution no.	Sn(II) salt	Ligand	Additive	% Sn(II)
1^†^	SnF_2_	–	–	7.49 ± 1.5
2	SnF_2_	PP	–	36.9 ± 0.1
3	SnF_2_	PP	KNO_3_	87.2 ± 0.1
4^†^	SnF_2_	–	KNO_3_	8.9 ± 0.5
5	SnF_2_	PP	NaNO_3_	85.7 ± 0.1
6	SnF_2_	PP	KCl	34.2 ± 0.1
7	SnCl_2_	PP	KNO_3_	78.9 ± 0.1
8	SnPP	PP	KNO_3_	83.6 ± 0.4
9	SnF_2_	EDTA	–	2.2 ± 0.8
10	SnF_2_	EDTA	KNO_3_	43.3 ± 2.6
11	SnF_2_	Citrate	–	2 ± 0.1
12	SnF_2_	Citrate	KNO_3_	51.5 ± 0.6

*The concentration of each material was 29 mM.

†Solutions forming a precipitate after addition. pH changes were minimal during the measurement period ~ΔpH = 0.1.

In addition, the effect of pH, [NO_3_^−^], and PP:Sn molar ratio on Sn(II) oxidation are presented in table S2. Specific variations within these results are attributed to competing chemical processes that affect Sn(II) oxidation, such as increased hydrolysis and changes to the SnF_2_ and PP [Sn(II)-PP] complex structure through increased ligand:Sn(II) ratio, or relevant p*K*_a_ of PP (where *K*_a_ is the acid dissociation constant). What is universally true is that the effect of NO_3_^−^ was observed under all of these conditions and at molar ratios as low as 0.01:1 NO_3_^−^:Sn(II)-PP. To that end, our subsequent investigations into the role of NO_3_^−^ focus on two systems: solutions containing SnF_2_ and PP [Sn(II)-PP] and SnF_2_, PP, and KNO_3_ [Sn(II)-PP-NO_3_].

### NMR spectroscopy

**Fig. 2.**
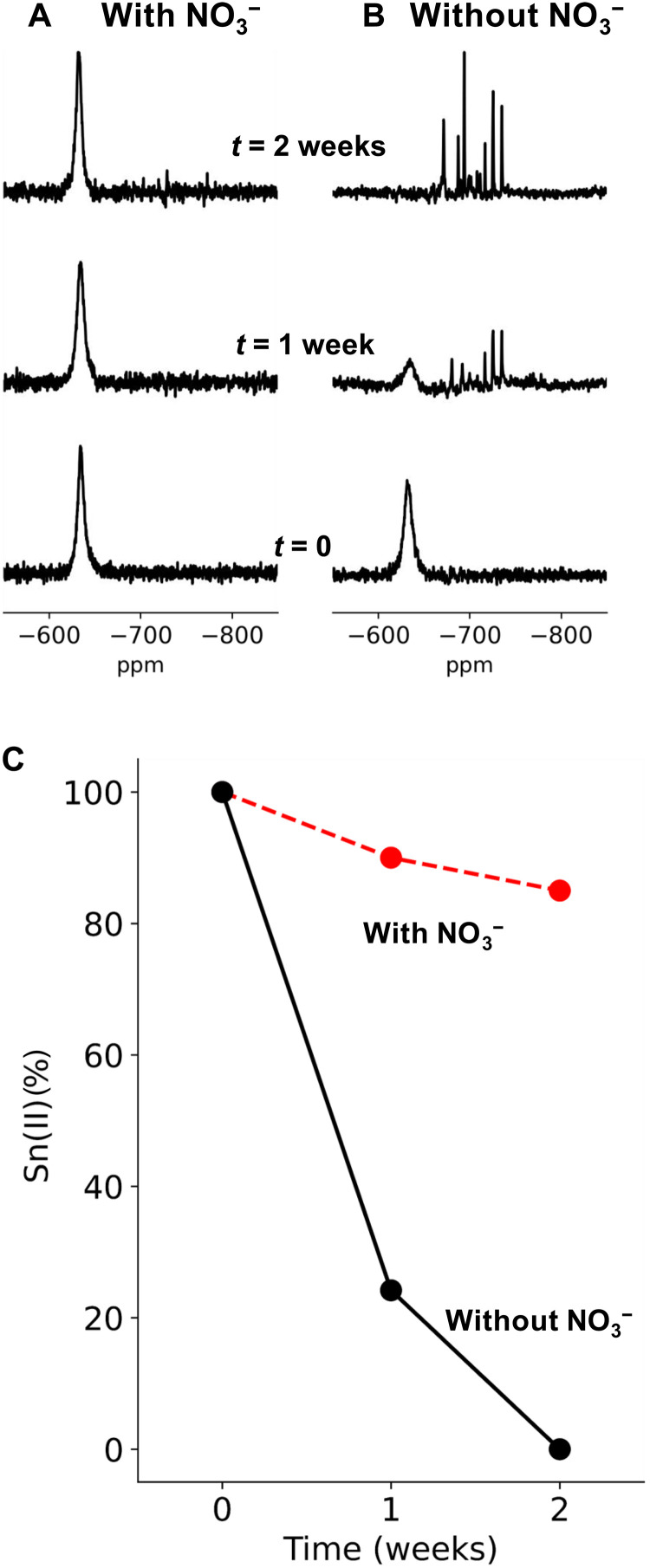
Evolution of ^119^Sn NMR spectra of chelated Sn(II) over time. (**A**) Sn(II)-PP-NO_3_, (**B**) Sn(II)-PP, and (**C**) relative Sn(II) concentration (%) estimated from Sn(II) peak integrations, taken at *t* = 0 weeks (bottom), *t* = 1 week (middle), and *t* = 2 weeks (top) of solutions held at 60°C. ppm, parts per million.

To understand how NO_3_^−^ influences the stability of Sn(II) at a molecular level, we acquired a series of ^119^Sn NMR spectra over time. Figure 2A shows spectra taken of solution Sn(II)-PP-NO_3_ with their peak integrals shown in Fig. 2C (red). In the presence of NO_3_^−^, the Sn(II) chemical shift remained unchanged, and the peak integral decreased only marginally to ~85% of its original value, consistent with the titrations described above. Sn(IV) complexes were not observed. In contrast, spectra for Sn(II)-PP (Fig. 2, B and C, black) show that [Sn(II)] diminished to near zero, with a simultaneous increase in numerous peaks attributed to Sn(IV) complexes of various configurations (*35*). Examinations of ^15^N and ^31^P within Sn(II)-PP-NO_3_ were also conducted. Specific to NO_3_^−^, ^15^N chemical shifts remained unchanged in the presence of Sn(II), indicating that NO_3_^−^ did not degrade or have substantial coordination with a Sn(II)-PP complex within these timescales (figs. S1 and S2).

### Sn(II) reactivity with molecular oxygen

Reactivity with molecular O_2_/ROS is a major Sn(II) oxidation pathway (*28*). To determine the degree to which NO_3_^−^ disrupts O_2_ reactivity specifically, we measured the aqueous concentration of O_2_ in various solutions (fig. S3). High [O_2_] in the presence of NO_3_^−^ would suggest interference with the Sn(II)-O_2_ reaction, provided that the Sn(II)-O_2_ reaction is faster than headspace O_2_ diffusion into the water solvent. In comparison to water and KNO_3(aq)_ controls, a solution of Sn(II)-PP was measured to have nearly completely consumed O_2_ over the duration of the experiment. In contrast, Sn(II)-PP-NO_3_ was observed to only partially consume O_2_.

**Fig. 3.**
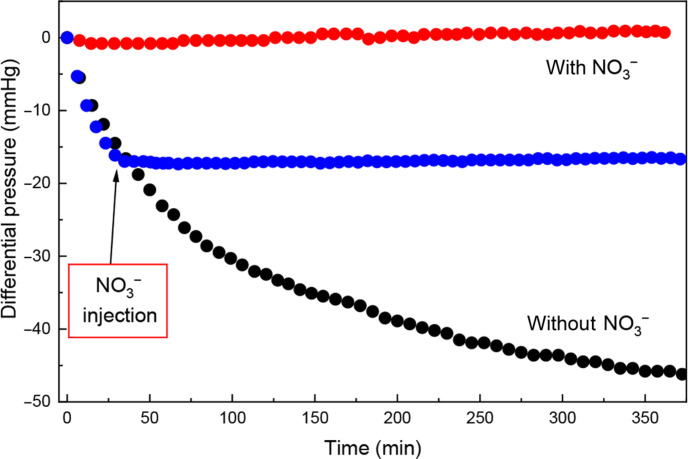
Headspace O_2_ consumption by Sn(II)-PP solutions with and without nitrate. Stannous oxidation reactions are monitored indirectly through differential pressure changes above solutions containing Sn(II)-PP with KNO_3_ (red), without KNO_3_ (black), and with a delayed addition of KNO_3_ (blue) (1:1 NO_3_^−^:Sn molar ratio), showing the suppression of Sn(II)-PP oxidation rate in the presence of nitrate ion.

Further to this point, we measured headspace O_2_ consumption in a closed vessel where the progression of the Sn(II)-O_2_ reaction was monitored indirectly through gas-phase pressure changes above the solution of interest. A depletion of O_2_, observed by a decrease in pressure, would indicate a Sn(II)-O_2_ reaction and Sn(II) oxidation. Figure 3 shows the differential pressure readings as a function of time for solutions of Sn(II)-PP and Sn(II)-PP-NO_3_. In the absence of NO_3_^−^, a continuous drop in pressure was observed over a 6-hour period, indicating a rapid consumption of O_2_ via the Sn(II) → Sn(IV) oxidation process (Fig. 3, black). In contrast, when NO_3_^−^ was present in the same solution, no substantial decrease in pressure was observed over the same period of time (Fig. 3, red). In a subsequent experiment, the addition of NO_3_^−^ was then delayed for 30 min (Fig. 3, blue). Before the addition, the reaction proceeded identically to Sn(II)-PP and consumed O_2_ as expected during this time. After NO_3_^−^ was injected, an immediate system response was observed, reducing the rate of O_2_ consumption.

### Mechanistic insights

To gain insight into this chemistry, we considered three different chemical causes: the role of reducing impurities within KNO_3_, changes to Sn(II) reactivity and oxidative kinetics as a result of NO_3_^−^ coordination, or that NO_3_^−^ reacts with tin and/or ROS, suggesting the presence of transient intermediate states. First, small concentrations of reducing impurities within NO_3_^−^ salts could act as sacrificial electron donors, helping to keep Sn(II) in a reduced state. This is unlikely, however, because the concentration of NO*_x_* or metal impurities is generally in a range of parts per billion and, if sacrificial, would be consumed very quickly. Oxidation rates would thus resemble Sn(II)-PP almost immediately, which, in our case, they do not.

Further to this point, we conducted an experiment identical to that in solution 3 in Table 1 with ultrahigh-purity SnCl_2_ and NaNO_3_. In this experiment, [Sn(II)] reduced by 22% in the presence of NO_3_^−^ versus ~50% in its absence, consistent with solution 3. Moreover, we examined the stability of Sn(II)-PP with a solution of KNO_3_ that had been bubbled in with air for 24 hours to convert any reducing impurities to a fully oxidized state. In this experiment, the data were equivalent to Fig. 3 with nitrate exhibiting the same suppressing effect on Sn(II) oxidation kinetics.

Second, changes in the oxidation potential of metal ions as a function of ligand have been well documented with examples of steric (*36*) and electronic (*37*) mechanisms being prevalent. Nitrate has a labile nature with poor binding affinity to metal centers (*38*). Studies on (CH_3_)Sn^2+^ in NO_3_^−^ solutions concluded that NO_3_^−^ had negligible effects on Sn-centered hydrolysis reactions (*39*). In our studies, a discrete complex of Sn(II)-PP with NO_3_^−^ was not observed through ^15^N NMR, ^119^Sn NMR, Fourier transform infrared, or mass spectrometry experiments (figs. S2 and S4).

A third possibility is that NO_3_^−^ acts as a radical scavenger or that it interferes with the Sn(II)-ROS reactions. Reactive oxygen species such as superoxide anions and hydroxyl radicals are short-lived species that are highly reactive and participate in a variety of chemical or biological reactions (*40*, *41*). To determine whether Sn(II) oxidation may proceed through a radical mechanism, we subjected solutions of Sn(II)-PP and Sn(II)-PP-NO_3_ to ultraviolet (UV) light radiation to generate ROS in situ (*40*). Through ^119^Sn NMR experiments (fig. S5), we observed an acceleration of Sn(II) oxidation in the absence of NO_3_^−^, suggesting the radical nature of the reaction. In agreement with thermally accelerated Sn(II) oxidation experiments, vide supra, the presence of NO_3_^−^ ion in Sn(II)-PP solution inhibited Sn(II) reactivity with oxygen species. To help identify oxidizing radicals and potential intermediate species, we turned to EPR spectroscopy.

**Fig. 4.**
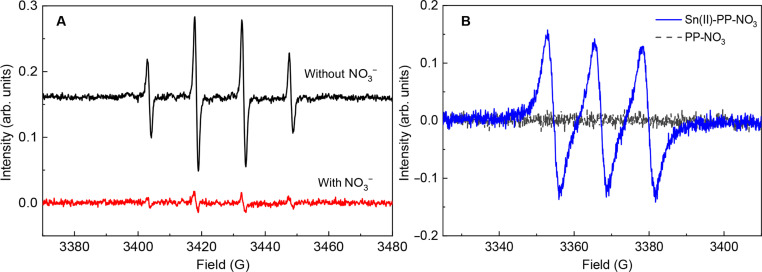
EPR analysis of reactive oxygen and nitrogen species. (**A**) EPR spectra of freshly prepared solutions containing Sn(II)-PP with and without KNO_3_. The spectra are offset for clarity. The hyperfine splitting constants are *a*_N_ = *a*_H_^β^ = 14.9 G, confirming the DMPO/·OH origin of the signal. (**B**) EPR spectrum of freshly prepared solution containing Sn(II)-PP-NO_3_ and (MGD)_2_-Fe(II) complex versus a control solution containing only PP and KNO_3_ with (MGD)_2_-Fe(II) complex. In presence of Sn(II), a characteristic triplet EPR signal of (MGD)_2_-Fe(II)-NO with *a*_N_ = 12.6 G is observed. Arb. units, arbitrary units.

Figure 4A displays the EPR spectra of the 5,5-dimethyl-1-pyrroline-*N*-oxide (DMPO)/·OH adduct in solutions of Sn(II)-PP and Sn(II)-PP-NO_3_ (see Materials and Methods for details). In the absence of NO_3_^−^, a distinct signal with characteristic 1:2:2:1 pattern was detected immediately in the freshly prepared solution. In the presence of NO_3_^−^ (Fig. 4A, red), the signal was suppressed, indicating a decreased [·OH], consistent with the data discussed above, and suggesting that NO_3_^−^ interferes with oxygen-based reaction pathways (see fig. S6 for more information).

We further used EPR spectroscopy to look for reactive nitrogen species such as N-based oxides (NO*_x_*) present in our samples (*42*, *43*). As shown in Fig. 4B, the signal attributed to (MGD)_2_–Fe(II)–NO adduct (MGD = N-methyl-d-glucamine dithiocarbamate) was observed in the solution containing Sn(II) and not in the solution of PP-NO_3_, in agreement with the previous report (*44*). Note here that specific NO*_x_* cannot be discerned with this method. The previous study has demonstrated that (MGD)_2_-Fe(II) is not specific to NO but can also generate the (MGD)_2_-Fe(II)-NO spin adducts in presence of NO_2_ and NO_2_^−^ (*44*).

Nitrate inhibition of Sn(II) oxidation appears efficient, such that we observed decreased Sn(II/IV) oxidation at low NO_3_^−^:Sn stoichiometric ratios, as low as 0.01:1 NO_3_^−^:Sn (table S2) (*45*). This concentration threshold may be a reflection of the natural concentration of ROS in solution, typically <10^−12^ M (*46*), below which ROS reactivity with Sn(II) may be faster than NO_3_^−^ diffusion to the Sn(II) metal center in aqueous solutions. In addition, the effects reported here are not absolute, as oxidation still occurs; NO_3_^−^ inhibits a major kinetically driven pathway, but not all. We did not observe a decrease in NO_3_^−^ concentration over the experimental window (Δ[NO_3_^−^] < 1% ± 3%), suggesting that the mechanism is at least partially cyclic in nature. This process also appears somewhat unique to Sn(II). We did examine the effect of NO_3_^−^ on Fe(II/III) oxidation because both metals undergo autoxidation (*47*–*49*). In the case of Fe, we did not observe nitrate to have any measurable effect (fig. S7).

Nitrate appears to have a pH-driven effect on ·OH and can act as both a scavenger (*50*, *51*) and a promoter (*46*). As a scavenger, previous authors proposed that NO_3_^−^ scavenges ·OH in acidic pH with a forward rate constant of 8.6 ± 1.3 × 10^7^ liters mol^−1^ s^−1^, forming H_2_O_2_ and NO_2_ products, which regenerate HNO_3_ under acidic conditions (*50*). In the present system, the near complete suppression of ·OH, the detection of NO*_x_*, and a sustained Sn(II) oxidation state indicate that a scavenging reaction is taking place and is one that happens at a pH substantially higher than reported previously. Because NO*_x_* was only observed in the presence of Sn(II)-PP, the reaction is clearly triggered by Sn(II).

Despite its heavy commercial use and with potential application as a nontoxic alternative metal (*10*), studies of aqueous Sn(II) are underreported in the recent literature. What appears to be universally accepted is that Sn(II) complexes behave in unexpected ways. Barnum (*52*) reported that Sn(II)_aq_ is unusually acidic, likely having too few water molecules in its first coordination sphere. In agreement, Persson *et al.* (*53*) surmised that occupation of an antibonding orbital may cause a void about the metal center. This theory is further supported by crystallography of [Sn(H_2_O)_3_]^2+^ where aqua ligands occupied ~15% of the coordination sphere (*54*). Rode and colleagues (*55*) revealed an unexpected discrepancy in water exchange rates between first and second coordination spheres, further describing Sn(II) as exhibiting behavior similar to monovalent cations than divalent (*52*, *55*, *56*). In our own studies, we observed a pH and PP:Sn ratio dependence on NO_3_^−^ stabilization of Sn(II)-PP (table S2). If aqua ligand exchange rates are a critical parameter to this mechanism, then a change in pH and higher PP:Sn ratios could affect Sn(II) complex speciation and could lead to a decreased NO_3_^−^ efficiency, similar to our observations.

Studies of PP complexes of Sn(II) are more limited still. The specific structure of the coordination complex adopts a variety of states within the same solution (*29*). In addition, the crystal structures of α- and β-Sn_2_P_2_O_7_ contain no water molecules, implying that unoccupied coordination sites may still play a role in PP-complexed Sn(II) (*31*). Further confounding is that this interaction is not limited to complexes of Sn(II) and PP. Nitrate also slowed oxidation rates in Sn(II)-citrate and Sn(II)-EDTA solutions (Table 1, table S2, and figs. S8 and S9), implying that Sn(II)_aq_ can be bound in a number of conformations and with different ligands (*57*).

This work demonstrates the radical nature of Sn(II)-PP stabilization by NO_3_^−^, with intermediates ·OH and NO*_x_* evident. The mechanism by which Sn(II) may contribute here is under investigation, although control experiments imply its involvement. We note that intermediate states of metals centers with apparent two-electron oxidations, such as Tl(I/III) (*58*) and Pb(II/IV) (*59*), have been studied and further proposed in the case of Sn (*48*, *60*). Because of its highly reactive nature and short lifetimes, direct observation of such intermediates has historically required experimental conditions that are well outside those presented here (*61*–*63*). We are continuing to explore the specifics of this reaction chemistry.

### Biological activity and efficacy

Pathogenic bacteria within the oral cavity are a major initiator of early and advanced staged oral health complications, with caries (*64*) and periodontal diseases (*65*) dominating severe global cases (*66*, *67*). As a result, therapeutic intervention with a chemotherapeutic agent, such as SnF_2_, is critical to control bioloads and maintain oral health. To that end, we examined the in vitro ability of Sn(II)-PP-NO_3_ to maintain antibacterial efficacy, reduce plaque bioload, and control inflammation to approximate its potential in vivo efficacy.

**Fig. 5.**
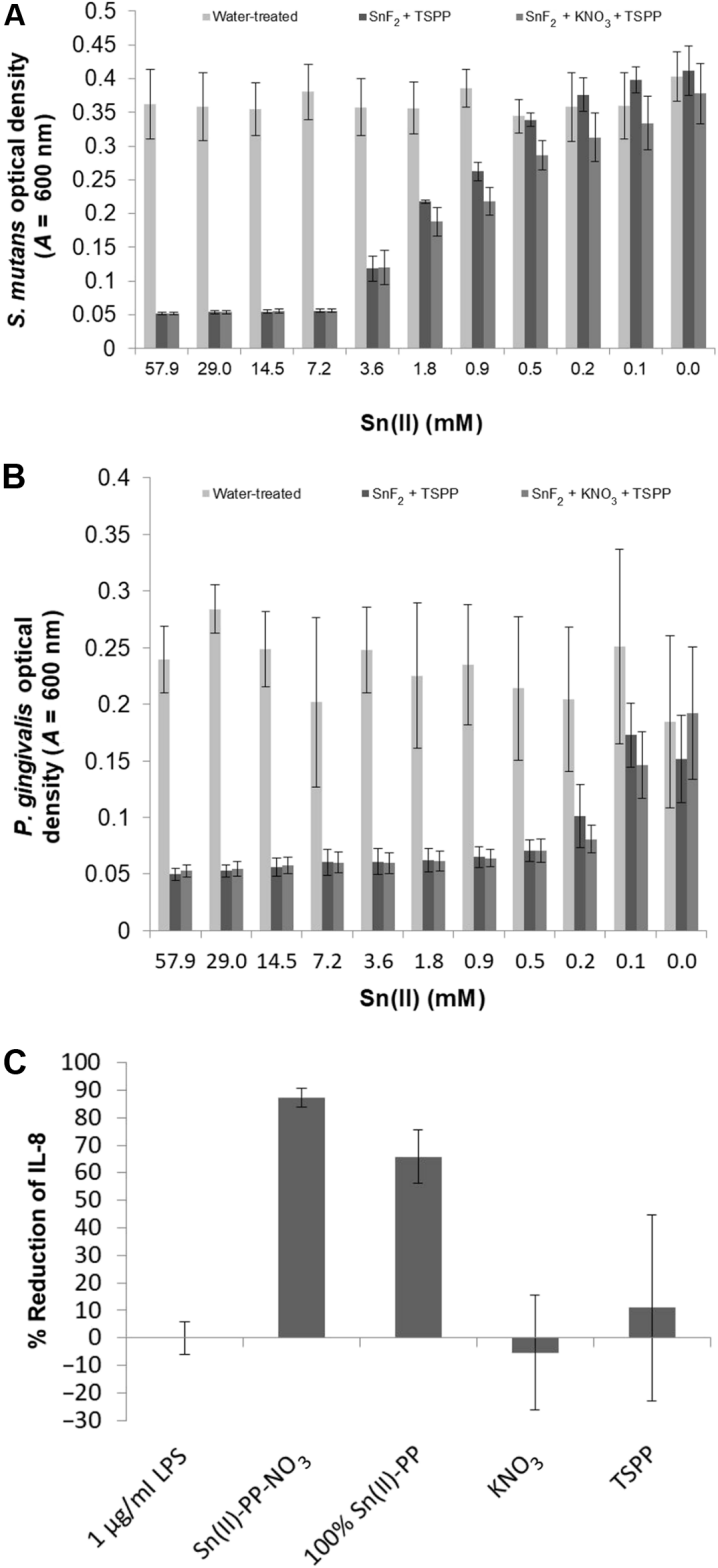
Impact of nitrate stabilization of Sn(II)-PP on antibacterial and anti-inflammatory efficacy. Growth inhibition studies using (**A**) *S. mutans* and (**B**) *P. gingivalis*. (**C**) Inflammation control on LPS-challenged HEK-hTLR4 cells as a function of Sn(II)-PP-NO_3_, Sn(II)-PP, and control compounds.

Culturing either *Porphyromonas gingivalis* or *Streptococcus mutans* in the presence of Sn(II)-PP or Sn(II)-PP-NO_3_ inhibited the growth of both pathogenic bacterial species relative to the water-treated negative control. This is consistent with the known antibacterial function of SnF_2_ at approximately 0.454 wt % (~29 mM) (*8*). Significant growth for *S. mutans* was not observed until SnF_2_ was diluted to approximately 0.9 mM (*P* ≤ 0.0013 for SnF_2_-PP and *P* ≤ 0.0001 for Sn(II)-PP-NO_3_; Fig. 5A) when compared against the water-treated negative control. Similarly, significant growth for the anaerobic periodontal pathogen, *P. gingivalis*, was not observed until SnF_2_ was diluted to approximately 0.5 mM for Sn(II)-PP (*P* ≤ 0.0056) and 0.1 mM for Sn(II)-PP-NO_3_ (*P* ≤ 0.0479) when compared with the water-treated group (Fig. 5B). These results indicate that the addition of NO_3_^−^ did not negatively affect the antibacterial activity of the metal salt, allowing it to maintain the potent broad-spectrum bactericidal function of Sn(II). The observed increase in antibacterial efficacy is attributed to an increased concentration of Sn(II).

These growth inhibition studies also showed that the addition of NO_3_^−^ with Sn(II)-PP maintained antibacterial performance of SnF_2_, namely, through enhanced stabilization of Sn(II) active species. Significantly less *S. mutans* growth was observed at 0.2 mM (*P* ≤ 0.0202) and 0.1 mM (*P* ≤ 0.0170) SnF_2_ in the Sn(II)-PP-NO_3_ treatment group than *S. mutans* treated with equivalent concentrations of Sn(II)-PP alone. Although not substantially reduced, a similar outcome was documented for *P. gingivalis* with directional improvement in efficacy in the Sn(II)-PP-NO_3_ treatment group at concentrations of 0.2 and 0.1 mM SnF_2_ when compared against the SnF_2_-PP treatment group. Our current data suggest that the use of NO_3_^−^ may enhance Sn(II) bioavailability, leading to an improvement in growth inhibition for the Sn(II)-PP-NO_3_ treatment group. These results are consistent with previous studies that indicated differences in the susceptibilities of different bacterial groups to therapeutic actives (*68*). While the mechanism (*69*) is still being explored for different microbial species, the current data show that SnF_2_ delivery in this way is a potent antimicrobial agent and can help in controlling plaque accumulation through continued use.

SnF_2_ has historically been shown to be effective in controlling gingival inflammation. Our current data are consistent with these findings and showed that the treatment groups containing Sn(II) dampened the induction of the proinflammatory cytokine, interleukin-8 (IL-8), as compared to the NO_3_^−^ or PP buffer negative controls (Fig. 5C). Treatment of *P. gingivalis* lipopolysaccharide (LPS)–challenged human embryonic kidney (HEK293)–human Toll-like receptor 4 (hTLR4) cells with either Sn(II)-PP or Sn(II)-PP-NO_3_ reduced IL-8 production of 65.8 and 85.3%, respectively, with minimal observed evidence of cytotoxicity. Relative to Sn(II)-PP–treated group, the Sn(II)-PP-NO_3_–treated cells showed significant reduction in IL-8 production (*P* ≤ 0.05), suggesting that maintaining the oxidation state of Sn(II) through the addition of NO_3_^−^ could result in an improvement in the metal ion’s antibacterial and anti-inflammatory functionality. Further to this point, clinical studies of dentifrices containing Sn(II)-PP-NO_3_ were shown to be efficacious at delivering SnF_2_ functionality (*70*, *71*).

In summary, the implementation of Sn(II) salts into a variety of fields has been challenging because it easily oxidizes under normal atmospheric conditions, resulting in loss of function. However, the combination of chelated Sn(II)_aq_ with low concentrations of NO_3_^−^ suppresses Sn(II) oxidation and does so in the presence of O_2_ across a broad pH range. Evidence of a radical nitrogen species without a decrease in NO_3_^−^ concentration suggests a cyclical radical–based mechanistic process. This unique chemical interplay between chelated Sn(II) and NO_3_^−^ lays the groundwork for additional chemistries reliant on the Sn(II) oxidation state and may improve processing or long-term functionality.

## MATERIALS AND METHODS

### Materials

Reagents SnF_2_, tetrasodium PP > 98%, EDTA > 99.4%, trisodium citrate dihydrate (citrate) > 99.0%, KNO_3_ > 99% purity, ultrahigh-purity NaNO_3_ (≥99.999%), and SnCl_2_ (≥99.9985%) were purchased from commercial sources, such as VWR. All reagents were used as received without further purification. Iodine (0.1 N) and sodium thiosulfate (0.1 N) solutions were purchased from VWR Chemicals BDH. D_2_O, spin trap DMPO (180 mM), iron(II) sulfate heptahydrate (FeSO_4_·7H_2_O), hemin, and menadione were purchased from Sigma-Aldrich. MGD sodium salt monohydrate was purchased from Enzo Life Sciences Inc. Dulbecco’s modified Eagle’s medium, fetal bovine serum, penicillin/streptomycin and PrestoBlue Cell Viability Reagent were purchased from Thermo Fisher Scientific. HEK-hTLR4 cells, *P. gingivalis* LPS, Normocin, and HEK-Blue selection media were purchased from Invivogen. The IL-8 enzyme-linked immunosorbent assay kit was purchased from Enzo Biochem. Tryptic Soy Broth (TSB) was purchased from BD Biosciences.

### Methods

NMR spectra were acquired using a Bruker AVANCE NEO 500 NMR spectrometer (Bruker, Billerica, MA, USA) with a liquid nitrogen cryogenic probe operating at 186.5 MHz at room temperature. Sn(II) oxidation was accelerated on static solutions using a Spectroline Hg lamp with short-wave UV (254 nm). EPR spectra were recorded on a Bruker EMXnano X-Band spectrometer. Headspace oxygen consumption measurements were conducted using a MP2000-002 manometer from APT Instruments. pH measurements were taken using a Thermo Fisher Scientific Orion Star A211 Benchtop pH meter. Titrations were performed using a BRAND Titrette digital bottle-top burette.

### Sn(II) solution preparation

Generally speaking, all solutions were prepared using identical methods to make 100 ml of solution volume with each included component at 29 mM concentration. When required, additives such as KNO_3_ and/or ligands such as PP were first dissolved in 90 ml of the deionized water (0.067 μS cm^−1^) by mechanical stirring, and once dissolved, SnF_2_ or alternative Sn(II) salts were then added. The solution was stirred until clear, and the pH was adjusted dropwise to ~7.0 using concentrated HCl or NaOH solutions. After pH adjustment, the remaining deionized water was added to bring a total solution volume of 100 ml, and the pH was measured again for confirmation. Before storing at 60°C, samples were sealed in 250-ml Nalgene plastic bottles and wrapped with Parafilm to minimize evaporation. Masses were recorded and evaluated, which ensured that evaporation did not take place during high-temperature storage conditions. Solutions of higher concentrations, such as those prepared for NMR and EPR experiments, presented a light suspension.

### Determination of Sn(II) through I_2_ titrations

Capitalizing on the previous report (*72*), a 10.00-g sample of a Sn-containing solution was weighed and fixed with a magnetic stir bar. If a precipitate was observed, then ~5 g of 2 M citric acid was added, and the resulting solution was stirred until the precipitate dissolved. Aliquots of 0.1 N of iodine were added in series until a persistent brown color was observed and the total volume added was recorded. To the resulting solution, 0.1 N of sodium thiosulfate was titrated slowly using a digital burette until the brown color dissipated. The volume of thiosulfate added was recorded, and Sn(II) was calculated by using a 1:1 molar ratio of Sn(II) reacting with I_2_ and 2:1 molar ratio of sodium thiosulfate reacting with I_2_. Data are reported as an average of *N* = 3.

### NMR spectroscopy

The samples were prepared and measured after the times and conditions indicated using aqueous solutions containing 10% (w/w) D_2_O, 128 mM SnF_2_ (to maximize signal response), 128 mM PP, and 128 mM KNO_3_ and adjusted to pH of 7.0 with NaOH. The ^119^Sn NMR chemical shift was determined relative to external tetramethyl tin. The relative Sn(II) concentration was estimated from the peak integral. The typical parameters were as follows: pulse width, 10 μs; recycle delay, 20 s; signal accumulation, 1024.

### Headspace O_2_ consumption

Using a similar reported procedure (*73*), stannous oxidation reactions were carried out in a closed 250-ml round-bottom flask and were monitored by pressure changes in the gaseous headspace above the solution at a constant temperature of 25° ± 0.5°C. In a typical experiment, the vessel was filled with 100 ml of solution containing 29 mM SnF_2_, 29 mM PP, and 29 mM KNO_3_. SnF_2_ was added last as a powder, and the flask was immediately sealed with a rubber septum and/or glass stopper. Presence of a septum allows injection of solution of interest at any time point of the reaction. The solution was constantly stirred magnetically, and the differential pressure was recorded over about a 6-hour period using a manometer connected to the flask. The pH of the solution was about 6.5 and did not substantially change during the reaction.

### EPR spectroscopy

Radicals of interest can be identified on the basis of their spectral line shapes and subsequent integration (*74*, *75*). For this purpose, special spin probe (i.e., spin trap) substances, for instance, DMPO, have been developed (*76*, *77*). DMPO is inherently EPR inactive but can react with a variety of free radicals, forming EPR-active adducts. Therefore, the quantification of radical generation (most commonly hydroxyl radical) in the sample with a unique spectral feature can be investigated. For ·OH radical detection, 5 μl of DMPO was added to a 250-μl aliquot of a pH ~6.5 aqueous solution containing 128 mM SnF_2_, 128 mM PP, and 128 mM KNO_3_. Twenty microliters of the resulting solution were transferred to a capillary tube that was then sealed with Sigillum Wax sealant (Globe Scientific Inc.) at both ends. The EPR room-temperature measurement conditions were as follows: frequency, 9.63 GHz; scan width, 150 G; power, 10 mW; receiver gain, 40 dB; modulation amplitude, 1 G; modulation frequency, 100 kHz; time constant, 1.28 ms; sweep time, 15 s; 300 scans. The samples were prepared in the dark (to avoid any exposure to UV light) and measured immediately after the preparation. For NO radical detection, stock solutions of MGD (100 mM) and FeSO_4_·7H_2_O (20 mM) were prepared in deoxygenated water and combined in equal volumes (25 μl) to form [Fe(MGD)_2_] complex in nitrogen-purged glove box. Fifty-microliter aliquot of a pH ~6.5 aqueous solution containing 128 mM SnF_2_, 128 mM PP, and 128 mM KNO_3_ (or just 128 mM PP and 128 mM KNO_3_, i.e., control sample) was added to (MGD)_2_-Fe(II) complex and vortexed. Twenty microliters of the resulting solution were transferred to a capillary tube that was then sealed with Sigillum Wax sealant at both ends. The EPR room-temperature measurement conditions were as follows: frequency, 9.63 GHz; scan width, 100 G; power, 17 mW; receiver gain, 20 dB; modulation amplitude, 1 G; modulation frequency, 100 kHz; time constant, 1.28 ms; sweep time, 30 s; 150 scans. All solutions were freshly prepared before the experiment (stannous solutions were prepared in the dark) and measured right after mixing.

### Bacterial growth inhibition assay

*S. mutans* (American Type Culture Collection, UA159) and *P. gingivalis* (American Type Culture Collection, 33277) were cultured respectively in either TSB at 37°C or TSB prepared with hemin and menadione at a final concentration of 5 and 1 μg/ml, respectively, at 37°C under anaerobic conditions. The bacterial cultures were then adjusted to an approximate optical density of ~0.05 (optical density = 610 nm) in their respective media before the start of the assay. Serially diluted Sn solutions were then seeded with equal volume (200-μl final volume) of bacterial suspensions and incubated anaerobically overnight at 37°C. The final optical density was then measured to assess for bacterial growth inhibition for each tested solution. The study was performed in triplicate experiments.

### Anti-inflammatory assessment

HEK-hTLR4 cells were grown until confluent and plated overnight in 96-well plates at 37°C and 5% CO_2_ with HEK-Blue selection media containing 10% fetal bovine serum, 1% penicillin/streptomycin, Normocin, and HEK-Blue selection. HEK-hTLR4 cells were coincubated overnight with ultrapure *P. gingivalis* LPS (1 μg/ml) and test solutions [2.9 mM SnF_2_, 1.71 mM PP, and/or 4.49 mM KNO_3_ (pH 7)]. The next day, cell culture supernatants were collected, and the level of proinflammatory marker IL-8 and cell viability were analyzed according to the manufacturer’s protocol. Statistical significance was determined on the basis of Student’s *t* test.

### Statistical analysis

A two-tailed *t* test was conducted to determine the significance of the cell viability and IL-8 expression on HEK-hTLR4 cells. Ninety-five percent was considered statistically significant. The untreated cells are used as control to compare to the cells treated with either ultrapure *P. gingivalis* LPS or/and test solutions [2.9 mM SnF_2_, 1.71 mM PP, and/or 4.49 mM KNO_3_ (pH 7)]. Bacterial growth inhibition statistical differences between each treatment group were determined using nonparametric two-way analysis of variance (ANOVA) using Dunnett’s multiple comparisons test.

## Supplementary Materials

### This PDF file includes:

Materials and Methods Figs. S1 to S9 Tables S1 and S2 References
