# Supplementary material for: Oxidative stability of chelated Sn(II)(aq) at neutral pH: The critical role of NO3− ions
Source: Sci Adv. 2024 Oct 2;10(40):eadq0839. doi: 10.1126/sciadv.adq0839 (PMC11446270; doi:10.1126/sciadv.adq0839)
Supplement: Supplementary file 1 — Materials and Methods Figs. S1 to S9 Tables S1 and S2 References [file sciadv.adq0839_sm.pdf]

Science Advances
AAAS

Supplementary Materials for

Oxidative stability of chelated $Sn(II)_{(aq)}$ at neutral pH: The critical role of NO_3^- ions

Shaoyi Zhang *et al.*

Corresponding author: Carl P. Myers, carl_myers@colpal.com; Tatiana V. Brinzari, tatiana_brinzari@colpal.com

Sci. Adv. **10**, eadq0839 (2024)
DOI: 10.1126/sciadv.adq0839

This PDF file includes:

Materials and Methods
Tables S1 and S2
Figs. S1 to S9
References

Methods and Materials

All chemicals and materials are the same as described in the main text. Experimental procedures specific to those listed here are as below:

Ion Chromatography: Nitrate concentration in solutions of Sn(II)-PP-NO_3, PP-NO_3 and KNO_3 was analyzed by gradient ion chromatography with suppressed conductivity detection (Dionex Capillary 4000 Ion Chromatographic System). Concentrations were determined by relating the nitrate peak area to the corresponding area of external calibration standard (sodium nitrate standard, chemical grade). Samples were prepared following the same procedure as described in the main text and aged at 60 °C for 2 weeks. The concentration of each ingredient was 29 mM.

NMR Spectroscopy. NMR spectra were acquired using a Bruker AVANCE NEO 500 NMR spectrometer (Bruker, Billerica, MA, USA) with a liquid nitrogen cryogenic probe operating at 186.5 MHz at room temperature. The samples were prepared and measured after the times and conditions indicated using aqueous solutions containing 10 % (w/w) D_2O, 128 mM SnF_2, 128 mM PP, and 128 mM KNO_3 (to maximize signal response), and adjusted to pH of 7.0 with NaOH. The ^{119}Sn NMR chemical shift was determined relative to external tetramethyl tin. The relative Sn(II) concentration was estimated from the peak integral. The typical parameters were as follows: pulse width: 10 μs, recycle delay 20 sec, signal accumulation: 1024.

Determination of Soluble O_2. Soluble oxygen measurements were performed with the HACH HQ30d dissolved oxygen (DO) meter. The samples were prepared as deionized aqueous solutions of 128 mM SnF_2, 128 mM PP, and 128 mM KNO_3, and adjusted to pH of 7.0 with NaOH. Water was used as a control to verify soluble oxygen readings. All of the solutions were measured at ambient room temperature conditions right after the preparation.

Fourier Transform Infrared Spectroscopy (FTIR): Infrared absorption spectra were collected using a Bruker Vertex 70 FTIR spectrometer (Bruker Optics, Billerica, MA) equipped with a GladiATR diamond ATR accessory (Pike technologies, Madison, WI). Spectra were recorded in the 80-4000 cm^{-1} spectral range at a resolution of 4 cm^{-1}. All measurements were carried out at room temperature on freshly prepared solutions. The concentration of each compound was 128 mM.

Headspace O2 consumption: Using a similar procedure as described in the main text, metal oxidation reactions were carried out in a closed 250 mL round-bottom flask and were monitored by pressure changes in the gaseous headspace above the solution at a constant temperature of 25 ± 0.5°C. In a typical experiment the vessel was filled with 100 mL of solution containing metal salt, chelant and KNO_3. Metal salt was added last as a powder, and the flask was immediately sealed with a rubber septum and/or glass stopper. The solution was constantly stirred magnetically and the differential pressure was recorded over time using a MP2000-002 manometer (APT Instruments) connected to the flask. The pH of the solutions was adjusted with NaOH/HCl to 6.5-7 and did not exhibit substantial changes during the reaction.

EPR Spectroscopy. Radicals of interest can be identified based on their spectral line shapes and subsequent integration (*74*, *75*). For this purpose, special spin probe (*i.e.*, spin trap) substances, for instance, 5,5 dimethyl-1-pyrroline N-oxide (DMPO), have been developed (*76*, *77*). DMPO is inherently EPR inactive but can react with a variety of free radicals, forming EPR-active adducts. Therefore, the quantification of radical generation (most commonly hydroxyl radical) in the sample with a unique spectral feature can be investigated.

OH radical detection: 5 μL of DMPO was added to a 250 μL aliquot of a pH ~6.5 aqueous solution containing 128 mM SnF_2, 128 mM PP, and 128 mM KNO_3. 20 μL of the resulting solution was transferred to a capillary tube that was sealed with Sigillum Wax sealant (Globe Scientific, Inc.) at both ends. EPR spectra were recorded on a Bruker EMXnano X-Band spectrometer. The EPR room-temperature measurement conditions were as follows: frequency, 9.63 GHz; scan width, 100 G; power, 10 mW; receiver gain, 40 dB; modulation amplitude 1 G; modulation

frequency 100 kHz; time constant, 1.28 ms, sweep time, 15 s, 25 scans. The samples were prepared in the dark and measured immediately after the preparation. After the acquisition of the first spectrum in the dark the samples were exposed to UV-VIS irradiation (100 Watt Hg UV lamp with a 380-560 nm bandpass filter) and recorded every 5 mins. The spectrum collection time was approximately 9 mins, so the reported light irradiation time is presented as an average of the starting and ending time. After signal acquisition, the spectra were plotted and analyzed using Origin 2018 software.

Table S1. General methods to improve Sn(II) efficacy through oxidative stability and solubility at physiological pH.

Method	Function	Shortcoming
Anhydrous manufacturing	Removal of water prevents hydrolysis	Expensive, difficult processing, compromised consumer attributes
Processing under N_2	Removes reactive O_2; Major oxidation pathway eliminated	Expensive, difficult processing, limited long-term storage stability
Ligand complexation	Increase solubility and/or oxidative stability	Limited effectiveness
Additional Sn(II) salts	Maintains high [Sn(II)]	Increased [M] is more astringent (taste compromise), increased tooth stain
Sacrificial reductants	Consumes Oxidant / Reduces Sn(IV) → Sn(II)	Limited effectiveness due to irreversibility of electron donation

Table S2. Percentage of Sn(II) remaining in aqueous solutions under various conditions.
Concentrations of PP and NO_3^- are indicated as molar ratios to SnF_2, prepared at 29 mM SnF_2. Variation to % Sn(II) indicates impact of changes in pH, [NO_3^-], and [PP] after storage at 60 ℃ for two weeks.

Sol #	[PP]	[NO_3^-]	pH[a]	% Sn(II)
1	1.05	-	4	2.4 ± 0.8
2	1.05	0.01	4	21.2 ± 0.5
3	1.05	0.1	4	38.7 ± 1.9
4	1.05	1	4	49.2 ± 2.3
5	1.05	-	6	35.3 ± 0.6
6	1.05	0.01	6	42.2 ± 0.5
7	1.05	0.1	6	69.4 ± 0.7
8	1.05	1	6	77.8 ± 0.5
9	1.05	-	8	1.1 ± 0.8
10	1.05	0.01	8	39.5 ± 0.5
11	1.05	0.1	8	39.2 ± 0.6
12	1.05	1	8	55.9 ± 0.4
13	1.5	1	7	87.1 ± 0.4
14	2.0	1	7	77.9 ± 0.3
15	3.0	1	7	72.6 ± 0.3

[a] pH changes were minimal during the measurement period ~ ΔpH = 0.1.

In order to expand the parameters at which NO_3^- impacts Sn(II) oxidation, we further examined the effect of pH, [NO_3^-], and Sn:PP ratio on Sn(II) oxidation with a series of solutions containing Sn(II)-PP; these data are shown in Table S1. Generally, the addition of NO_3^- decreased Sn(II) oxidation across the full pH range tested, and the effect was observed as low as 0.01:1 NO_3^-:Sn(II)-PP. We did observe a pH dependence where solutions with a pH of 6 or 7 exhibited the highest Sn(II) % while pH 4 and 8 were the lowest. While the effect of NO_3^- is clear, this pH-dependence can be at least partially attributed to competing chemical processes with the Sn-PP complex itself. As stated above, chelation of Sn(II) is a critical first step in maintaining its oxidation state. At lower pH, Sn-PP coordination is likely weakened, and thus destabilized, due to protonation of PP (pKa ~ 6), while at pH 8, Sn(II) hydrolysis reactions have increased competition. We also note that increasing [PP] led to a slight increase in Sn(II) oxidation, suggesting that over-chelation of Sn(II) hinders NO_3^- effects.

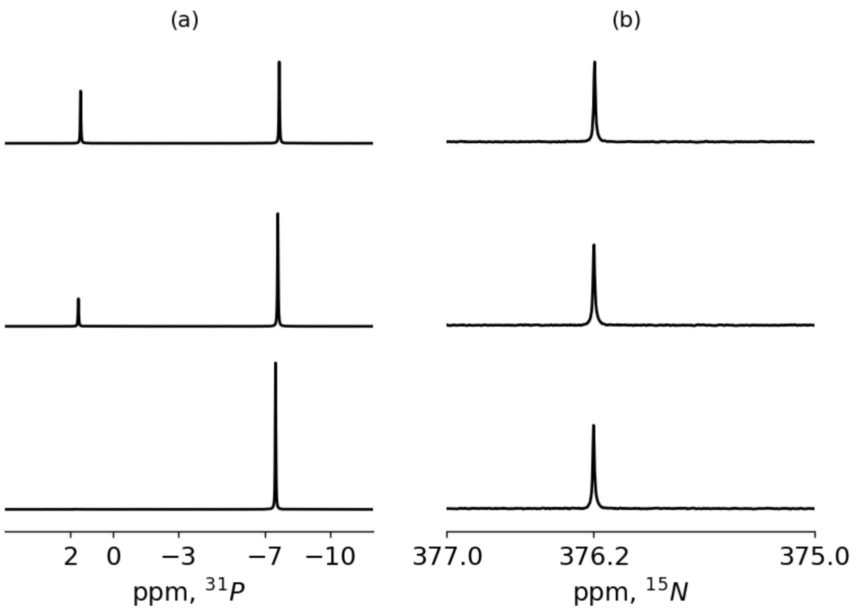

Fig. S1. ^{31}P and ^{15}N NMR spectra of Sn(II)-PP-NO_3 taken over time.
(a) ^{31}P NMR, (b) ^{15}N NMR of Sn(II)-PP-NO_3. Spectra were collected after solutions were stored at 60 ºC, taken at t = 0 (bottom), 1 week (middle), and 2 weeks (top). Over these conditions, a portion of the pyrophosphate has hydrolyzed to ortho-phosphate (*78–80*).

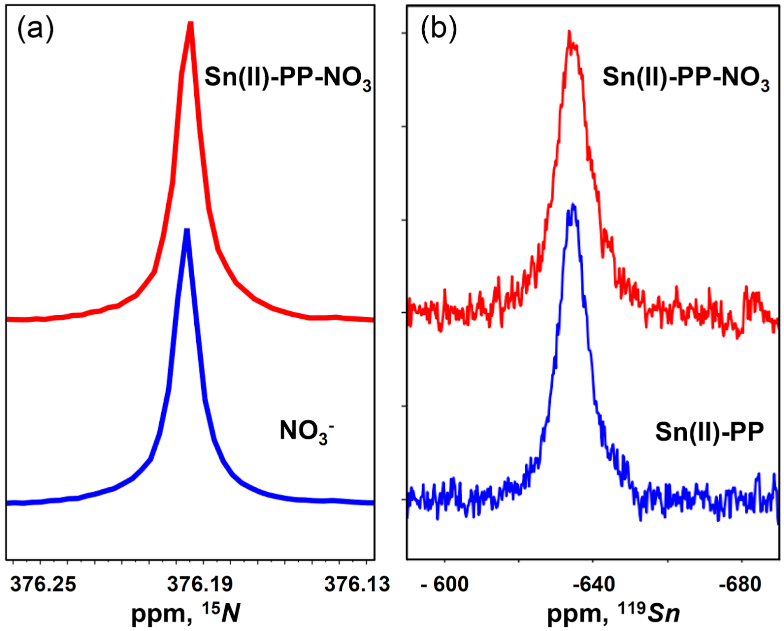

Fig. S2. ^{15}N and ^{119}Sn NMR spectra of Sn(II)-PP-NO_3, Sn(II)-PP, and nitrate.
(a) Comparison of ^{15}N NMR spectrum of $K^{15}NO_3$ solution with the spectrum of Sn(II)-PP-$^{15}NO_3$ sample. (b) ^{119}Sn NMR spectra of Sn(II)-PP solution with and without KNO_3. No difference in lineshape or peak positions are observed among tested solutions, and no apparent formation of mixed ligand Sn-PP-NO_3 complex is evident in either ^{15}N NMR or ^{119}Sn NMR spectra. The spectra are offset for clarity.

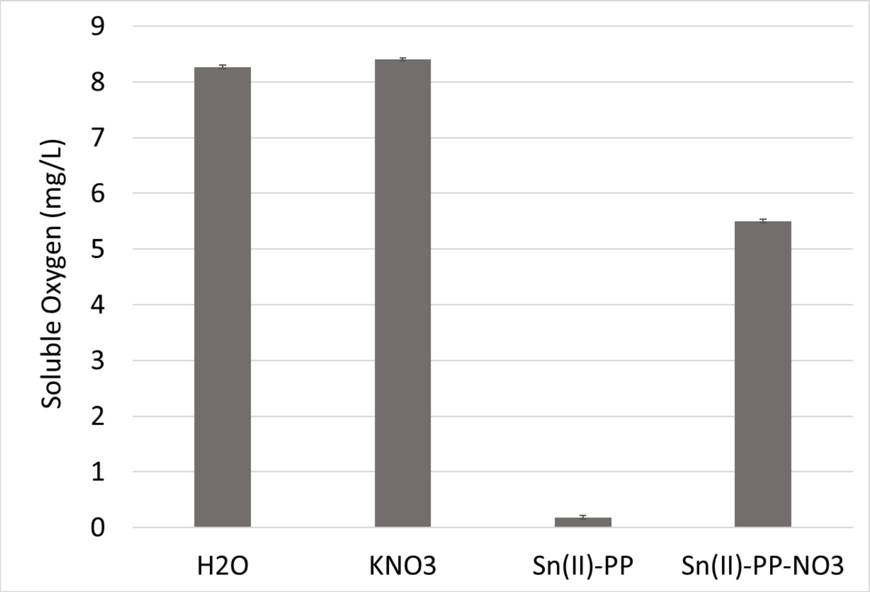

Fig. S3. Effect of NO_3^- on soluble oxygen in solutions of Sn(II)-PP.
Controls of H_2O and KNO_3 demonstrate soluble O_2 is depleted by Sn(II)-PP.

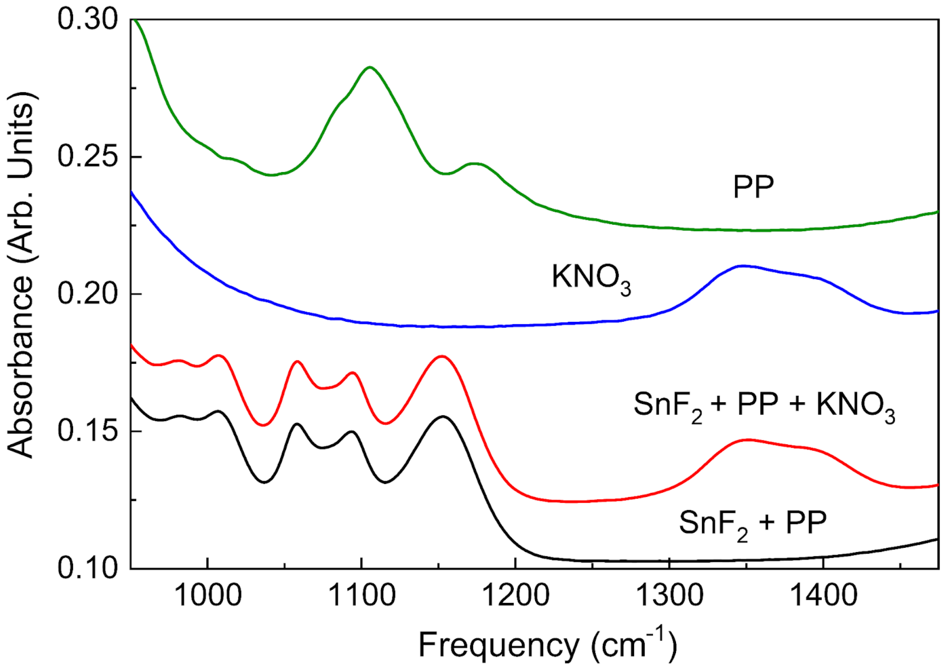

Fig. S4. Infrared absorption spectra of Sn(II)-PP solutions and controls.
The data clearly indicate the formation of tin pyrophosphate complex in presence of PP and no apparent complexation of tin with NO_3^- as the lineshape and peak positions of KNO_3 bands are the same with or without tin ion. The spectra are offset for clarity.

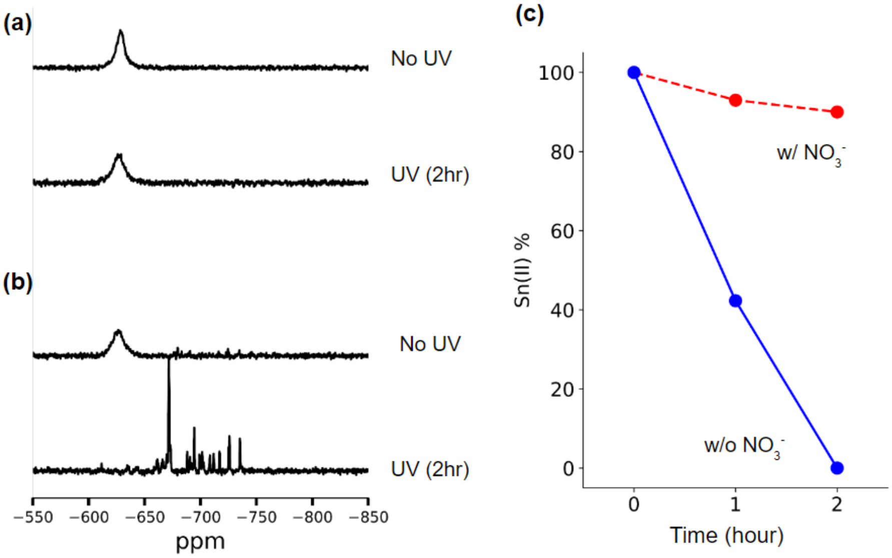

Fig. S5. ^{119}Sn NMR spectra and relative Sn(II) concentration in UV-aged Sn(II)-PP solutions.
(a) Sn(II)-PP-NO_3, (b) Sn(II)-PP, and (c) their Sn(II) integrated area before and after 2 hours of UV irradiation (Spectroline Hg lamp with 254 nm short wave UV). As a complementary data set to that shown in Fig. 1, these demonstrate that UV-irradiation has similar but accelerated oxidative results as thermally induced processes. The lines in (c) guide the eye.

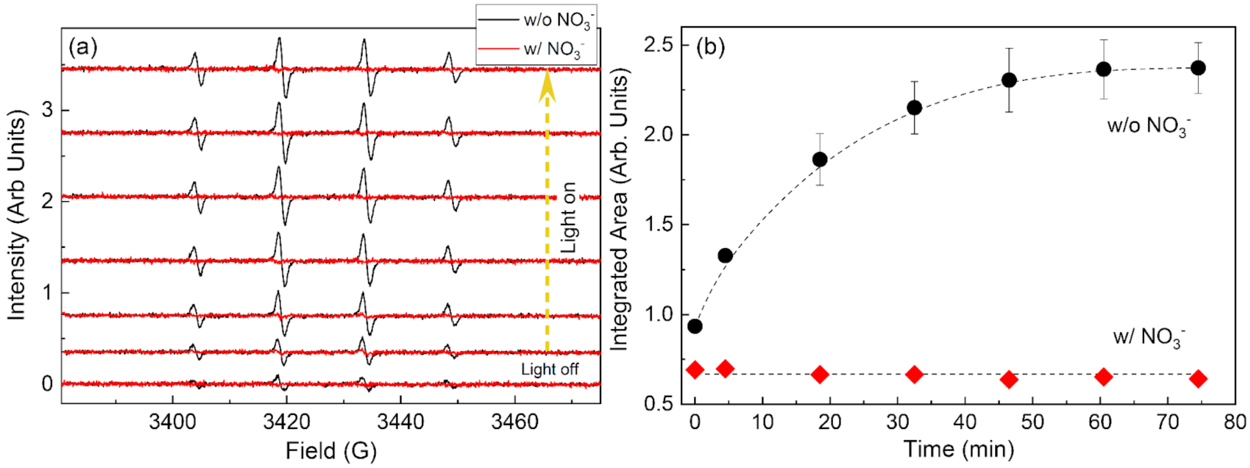

Fig. S6. Hydroxyl radical generation in Sn(II)-PP solutions under UV-VIS irradiation.
(a) EPR spectra of freshly prepared solutions containing SnF_2 and PP with and without KNO_3 measured in the dark (t=0 mins) and under the UV-VIS irradiation. The spectra are offset for clarity. (b) Integrated area of the EPR spectra (DMPO/•OH adduct signal range) for the Sn(II)-PP and Sn(II)-PP-NO_3 solutions clearly showing the suppression of hydroxyl radicals formation in the presence of KNO_3. The dashed lines guide the eye. Note, at long UV-VIS exposures spin trap will eventually start to degrade.

As discussed in the main text (Fig. 4A) hydroxyl radicals were detected in solutions of Sn(II)-PP and Sn(II)-PP-NO_3, with the DMPO/•OH adduct signal being suppressed in the presence of NO_3^-. We further explored this behavior by exposing the samples to UV-VIS irradiation (Fig. S6a). The total signal generated during this time was integrated and plotted for comparison in Fig. S6(b). Consistent with Fig. 4A, in the absence of NO_3^-, at t=0 min, we observed small but detectable levels of •OH radical, indicating that it was already present even in the freshly prepared samples. Upon UV-VIS irradiation the EPR signal rapidly increased and then plateaued. In stark contrast, in the presence of NO_3^-, the EPR signal was negligible, and remained flat throughout the measurement time further demonstrating the profound effect of nitrate ion on the oxidation rates of stannous in Sn(II)-PP complex.

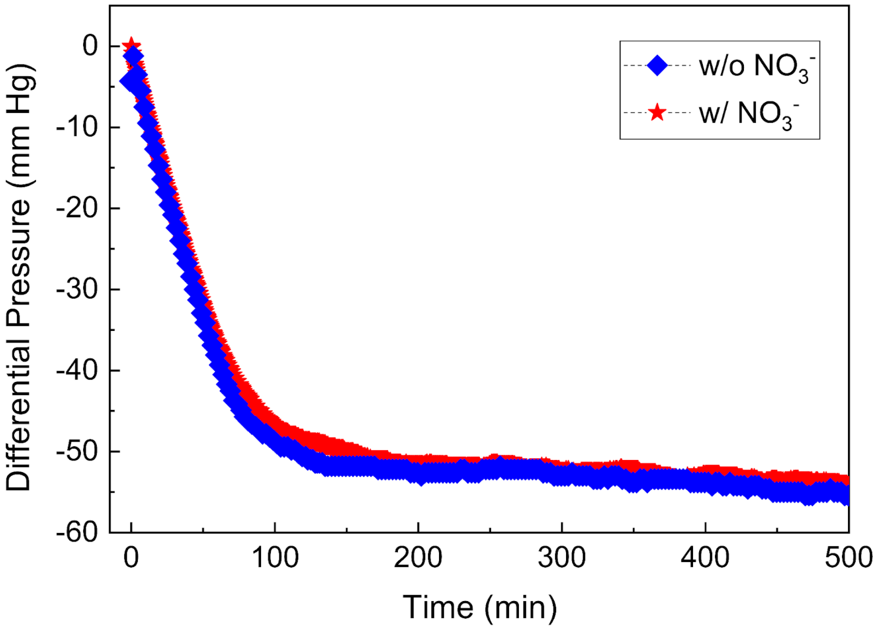

Fig. S7. Headspace differential pressure readings as a function of time for solutions containing $FeSO_4$ and PP with and without NO_3^-.

No effect of nitrate ion on iron(II) oxidation kinetics was observed. The concentration of each compound is 29 mM.

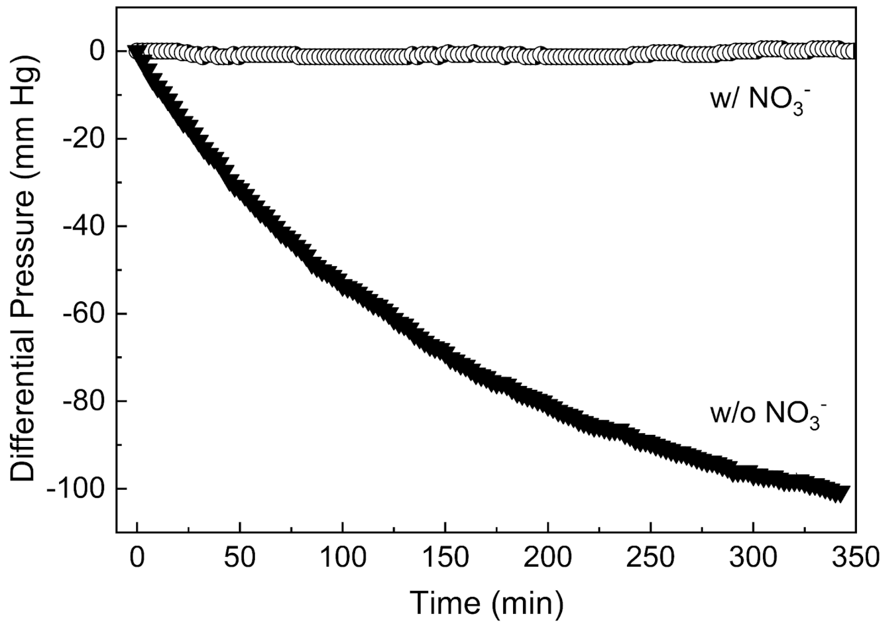

Fig. S8. Headspace differential pressure readings as a function of time in Sn(II)-citrate solutions.
Solutions contain 29 mM SnF_2 and 58 mM $HOC(COONa)(CH_2COONa)_2{\cdot}2H_2O$ with and without 29 mM KNO_3. The data clearly demonstrate a suppression of Sn(II)-citrate complex oxidation in presence of nitrate ion.

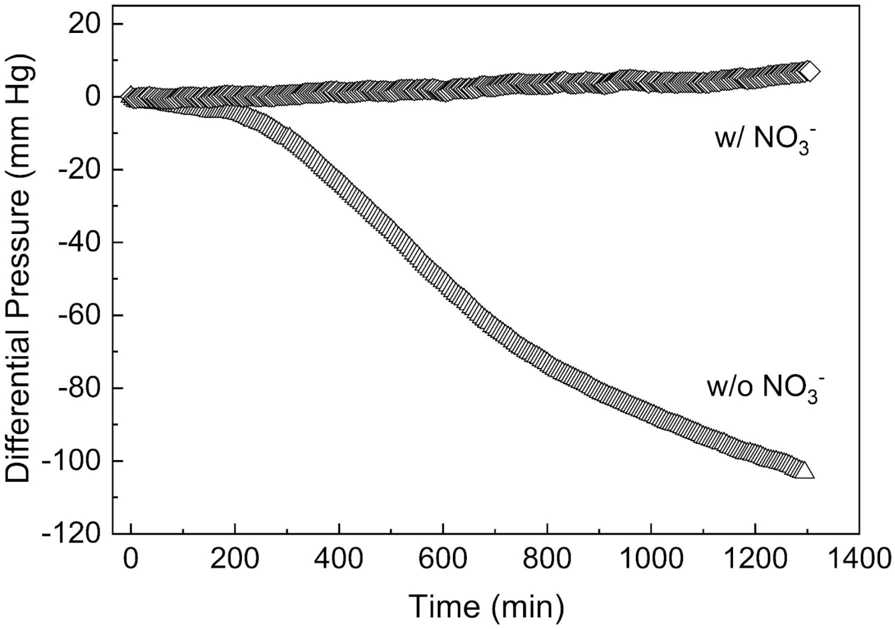

Fig. S9. Headspace differential pressure readings as a function of time in Sn(II)-EDTA solutions.
Solutions containing SnF_2 and ethylenediaminetetraacetic acid (EDTA) with and without KNO_3. A clear inhibition of Sn(II)-EDTA complex oxidation in presence of nitrate ion is observed. The concentration of each compound is 29 mM.

REFERENCES AND NOTES

1. N. Iwasawa, T. Yura, T. Mukaiyama, Catalytic use of tin(II) reagents in organic synthesis. *Tetrahedron* **45**, 1197–1207 (1989).

2. R. F. Storey, J. W. Sherman, Kinetics and mechanism of the stannous octoate-catalyzed bulk polymerization of ε-caprolactone. *Macromolecules* **35**, 1504–1512 (2002).

3. K. Stefaniak, A. Masek, Green copolymers based on poly(lactic acid)-short review. *Materials* **14**, 5254 (2021).

4. W. Han, M. Zhang, Y. Kong, D. Li, L. Liu, S. Tang, J. Ding, S. Liu, Pentaerythritol stearate ester-based tin (II) metal alkoxides: A tri-functional organotin as poly (vinyl chloride) thermal stabilizers. *Polym. Degrad. Stab.* **175**, 109129 (2020).

5. Y. G. Shi, Z. C. Li, L. Mei, P. Sun, The stannous complex of succinimide as multifunctional additive for lubricating oils. *Adv. Mat. Res.* **535-537**, 2479–2482 (2012).

6. E. Gundogdu, E. S. Demir, E. Özgenç, G. Yeğen, B. Aksu, Applying quality by design principles in the development and preparation of a new radiopharmaceutical: Technetium-99m-imatinib mesylate. *ACS Omega* **5**, 5297–5305 (2020).

7. N.-U.-H. Khan, S. A. R. Naqvi, H. Sohail, S. Roohi, M. A. Jamal, Technetium-99m labeled ibuprofen: Development and biological evaluation using sterile inflammation induced animal models. *Mol. Biol. Rep.* **46**, 3093–3100 (2019).

8. Food Drug Administration, "*Oral health care drug products for over-the-counter human use; antigingivitis/antiplaque drug products; establishment of a monograph*," 29 May 2023; www.govinfo.gov/content/pkg/FR-2003-05-29/pdf/FR-2003-05-29.pdf.

9. L. Chen, C. Hou, Z. Liu, Y. Qu, M. Xie, W. Han, Inhibition of Sn(II) oxidation in *Z*-scheme $BiVO_4$-QD@Sn_3O_4 for overall water splitting. *Chem. Commun.* **56**, 13884–13887 (2020).

10. W. Ke, M. G. Kanatzidis, Prospects for low-toxicity lead-free perovskite solar cells. *Nat. Commun.* **10**, 965 (2019).

11. S. Gupta, D. Cahen, G. Hodes, How SnF_2 impacts the material properties of lead-free tin perovskites. *J. Phys. Chem. C* **122**, 13926–13936 (2018).

12. E. W.-G. Diau, E. Jokar, M. Rameez, Strategies to improve performance and stability for tin-based perovskite solar cells. *ACS Energy Lett.* **4**, 1930–1937 (2019).

13. J. Liu, H. Yao, S. Wang, C. Wu, L. Ding, F. Hao, Origins and suppression of Sn(II)/Sn(IV) oxidation in tin Halide perovskite solar cells. *Adv. Energy Mater.* **13**, 2300696 (2023).

14. J. Pascual, G. Nasti, M. H. Aldamasy, J. A. Smith, M. Flatken, N. Phung, D. Di Girolamo, S.-H. Turren-Cruz, M. Li, A. Dallmann, R. Avolio, A. Abate, Origin of Sn(II) oxidation in tin halide perovskites. *Mater. Adv.* **1**, 1066–1070 (2020).

15. M. Hu, G. Wang, Q. Zhang, J. Gong, Z. Xing, J. Gao, J. Wang, P. Zeng, S. Zheng, M. Liu, Y. Zhou, S. Yang, Antioxidative solution processing yields exceptional Sn(II) stability for sub-1.4 eV bandgap inorganic perovskite solar cells. *J. Mater. Chem. A Mater. Energy Sustain.* **72**, 487–494 (2022).

16. C. P. Myers, I. Pappas, E. Makwana, R. Begum-Gafur, N. Utgikar, M. A. Alsina, M. Fitzgerald, H. M. Trivedi, J.-F. Gaillard, J. G. Masters, R. J. Sullivan, Solving the problem with stannous fluoride: Formulation, stabilization, and antimicrobial action. *J. Am. Dent. Assoc.* **150**, S5–S13 (2019).

17. J. C. Muhler, A. W. Radike, W. H. Nebergall, H. G. Day, The effect of a stannous fluoride-containing dentifrice on caries reduction in children. *J. Dent. Res.* **33**, 606–612 (1954).

18. J. C. Muhler, A. W. Radike, W. H. Nebergall, H. G. Day, Effect of a stannous fluoride-containing dentifrice on caries reduction in children II. Caries experience after one year. *J. Am. Dent. Assoc.* **50**, 163–166 (1955).

19. G. A. Nevitt, D. H. Witter, W. D. Bowman, Topical applications of sodium fluoride and stannous fluoride. *Public Health Rep.* **73**, 847–850 (1958).

20. D. J. White, A “return” to stannous fluoride dentifrices. *J. Clin. Dent.* **6**, 29–36 (1995).

21. A. Lussi, B. Megert, D. Eggenberger, T. Jaeggi, Impact of different toothpastes on the prevention of erosion. *Caries Res.* **42**, 62–67 (2008).

22. A Group of Experts, Clinical effectiveness of some fluoride-containing toothpastes. *Bull. World Health Organ.* **60**, 633–638 (1982).

23. L. T. Zhuravlev, The surface chemistry of amorphous silica. Zhuravlev model. *Colloids Surf. A: Physicochem. Eng. Asp.* **173**, 1–38 (2000).

24. M. Pettine, F. J. Millero, G. Macchi, Hydrolysis of tin(II) in aqueous solutions. *Anal. Chem.* **53**, 1039–1043 (1981).

25. N. Tinanoff, Review of the antimicrobial action of stannous fluoride. *J. Clin. Dent.* **2**, 22–27 (1990).

26. M. Desmau, M. A. Alsina, J.-F. Gaillard, XAS study of Sn speciation in toothpaste. *J. Anal. At. Spectrom* **36**, 407–415 (2021).

27. D.-R. Yang, Z.-L. Wu, K. Ren, P. Dong, D. Zhang, B. Yang, F. Liang, Recent advances of the thermodynamic behavior of tin species in aqueous solution. *J. Min. Metall. Sect. B* **59**, 1–15 (2023).

28. G. Denes, G. Lazanas, Oxidation of SnF_2 stannous fluoride in aqueous solutions. *Hyperfine Interact.* **90**, 435–439 (1994).

29. J. R. Duffield, D. R. Williams, I. Kron, Speciation studies of the solubility and aqueous solution chemistry of tin(II)- and tin(IV)-pyrophosphate complexes. *Polyhedron* **10**, 377–387 (1991).

30. N. X. West, T. He, Y. Zou, J. DiGennaro, A. Biesbrock, M. Davies, Bioavailable gluconate chelated stannous fluoride toothpaste meta-analyses: Effects on dentine hypersensitivity and enamel erosion. *J. Dent.* **105**, 103566 (2021).

31. M. R. Buchner, F. Kraus, H. Schmidbaur, Pyrophosphate complexation of tin(II) in aqueous solutions as applied in electrolytes for the deposition of tin and tin alloys such as white bronze. *Inorg. Chem.* **51**, 8860–8867 (2012).

32. S. Glodowski, Z. Kublik, Polarographic investigation of the stability of tin(II) solutions in the presence of some stabilizing agents. *Anal. Chim. Acta* **130**, 133–140 (1981).

33. N. Tinanoff, Progress regarding the use of stannous fluoride in clinical dentistry. *J. Clin. Dent.* **6**, 37–40 (1995).

34. R. K. Hart, The thermal oxidation of tin. *Proc. Phys. Soc. B* **65**, 955 (1952).

35. L. Mathieu, P. Thivolle, J. C. Duplan, M. Berger, ^{119}Sn NMR spectroscopy as evidence for a Pyr_2-Sn(IV) complex formation in mixed na pyrophosphate and stannous chloride aqueous solutions. *J. Magn. Reson.* **69**, 362–366 (1986).

36. M. T. Youinou, R. Ziessel, J. M. Lehn, Formation of dihelicate and mononuclear complexes from ethane-bridged dimeric bipyridine or phenanthroline ligands with copper(I), cobalt(II), and iron(II) cations. *Inorg. Chem.* **30**, 2144–2148 (1991).

37. J. M. Hudson, G. W. Luther III, Y.-P. Chin, Influence of organic ligands on the redox properties of Fe(II) as determined by mediated electrochemical oxidation. *Environ. Sci. Technol.* **56**, 9123–9132 (2022).

38. T. Gajda, J. Sangster, S. K. Saxena, W. Voigt, Chemical thermodynamics of tin. *Chem. Thermodyn.* **12**, 99206-1 (2012).

39. C. De Stefano, C. Foti, A. Gianguzza, M. Martino, L. Pellerito, S. Sammartano, Hydrolysis of $(CH_3)_2Sn^{2+}$ in different ionic media: Salt effects and complex formation. *J. Chem. Eng. Data* **41**, 511–515 (1996).

40. L. Zastrow, O. Doucet, L. Ferrero, N. Groth, F. Klein, D. Kockott, J. Lademann, Free radical threshold value: A new universal body constant. *Skin Pharmacol. Physiol.* **28**, 264–268 (2015).

41. N. A. Daugherty, J. H. Niewahner, Oxidation of tin(II) by hydrogen peroxide. *Inorg. Chem.* **11**, 535–537 (1972).

42. C. S. Lai, A. M. Komarov, Spin trapping of nitric oxide produced in vivo in septic-shock mice. *FEBS Lett.* **345**, 120–124 (1994).

43. J. L. Zweier, P. Wang, P. Kuppusamy, Direct measurement of nitric oxide generation in the ischemic heart using electron paramagnetic resonance spectroscopy. *J. Biol. Chem.* **270**, 304–307 (1995).

44. K. Hiramoto, S. Tomiyama, K. Kikugawa, Appearance of electron spin resonance signals in the interaction of dithiocarbamate-Fe(II) with nitrogen dioxide and nitrite. *Free Radic. Res.* **27**, 505–509 (1997).

45. S. Zhang, S. Tang, C. Myers, G. Xu, Oral care compositions and methods, US Patent US10918580B2 (2021).

46. S. Gligorovski, R. Strekowski, S. Barbati, D. Vione, Environmental implications of hydroxyl radicals (OH). *Chem. Rev.* **115**, 13051–13092 (2015).

47. K. D. Welch, T. Z. Davis, S. D. Aust, Iron autoxidation and free radical generation: Effects of buffers, ligands, and chelators. *Arch. Biochem. Biophys.* **397**, 360–369 (2002).

48. T. D. Smith, The effect of nitrate on the autooxidation of tin(II) in citric acid solutions. *Aust. J. Chem.* **20**, 15–19 (1967).

49. F. M. El-Demerdash, M. I. Yousef, M. A. Zoheir, Stannous chloride induces alterations in enzyme activities, lipid peroxidation and histopathology in male rabbit: Antioxidant role of vitamin C. *Food Chem. Toxicol.* **43**, 1743–1752 (2005).

50. S. I. Nikitenko, L. Venault, P. Moisy, Scavenging of $OH^{\cdot}$ radicals produced from H_2O sonolysis with nitrate ions. *Ultrason. Sonochem.* **11**, 139–142 (2004).

51. G. A. Poskrebyshev, P. Neta, R. E. Huie, Equilibrium constant of the reaction $\cdot OH + HNO_3$ □ $H_2O + NO_3$ in aqueous solution. *J. Geophys. Res.* **106**, 4995–5004 (2001).

52. D. W. Barnum, Hydrolysis of cations. Formation constants and standard free energies of formation of hydroxy complexes. *Inorg. Chem.* **22**, 2297–2305 (1983).

53. I. Persson, P. D'Angelo, D. Lundberg, Hydrated and solvated tin(II) Ions in solution and the solid state, and a coordination chemistry overview of the $d^{10}s^2$ metal ions. *Chemistry* **22**, 18583–18592 (2016).

54. E. Hennings, H. Schmidt, M. Köhler, W. Voigt, Crystal structure of tin(II) perchlorate trihydrate. *Acta Crystallogr. Sect. E Crystallogr. Commun.* **70**, 474–476 (2014).

55. T. S. Hofer, A. B. Pribil, B. R. Randolf, B. M. Rode, Structure and dynamics of solvated Sn(II) in aqueous solution: An ab initio QM/MM MD approach. *J. Am. Chem. Soc.* **127**, 14231–14238 (2005).

56. G. Johansson, H. Ohtaki, An x-ray investigation of the hydrolysis products of tin(II) in solution. *Acta Chem. Scand.* **27**, 643–660 (1973).

57. T. Brinzari, C.-Y. Cheng, Z. Hao, L. Pan, C. Castro, V. Dubovoy, Oral care compositions containing stannous ion source, US Patent US20240180799A1(2024).

58. D. E. Khoshtariya, T. D. Dolidze, L. D. Zusman, G. Lindbergh, J. Glaser, Two-electron transfer for $Tl(aq)^{3+}/Tl(aq)^{+}$ revisited. Common virtual $[Tl^{II}\text{-}Tl^{II}]^{4+}$ intermediate for homogeneous (superexchange) and electrode (sequential) mechanisms. *Inorg. Chem.* **41**, 1728–1738 (2002).

59. H. Liu, A. M. Kuznetsov, A. N. Masliy, J. F. Ferguson, G. V. Korshin, Formation of Pb(III) intermediates in the electrochemically controlled Pb(II)/PbO_2 system. *Environ. Sci. Technol.* **46**, 1430–1438 (2012).

60. W. C. E. Higginson, R. T. Leigh, R. Nightingale, 82. Reducing reactions of tin(II) in aqueous solution. Part I. A method for the detection of tin(III). *J. Chem. Soc.* **14**, 435–439 (1962).

61. N. Shinohara, M. Inoue, Photochemical generation of Sn(III) in hydrochloric acid solutions. *Bull. Chem. Soc. Jpn.* **62**, 730–733 (1989).

62. J. T. Yu, S. Lou, Y. Jeng, EPR of $[N(CH_3)_3]^+$ and $[SnX_6]$su3− (X= Cl, Br) in irradiated $[NH(CH_3)_3]_2SnX_6$ crystals. *J. Phys. Chem. Solid* **54**, 57–64 (1993).

63. J. Chang, A. J. Bard, Detection of the Sn(III) intermediate and the mechanism of the Sn(IV)/Sn(II) electroreduction reaction in bromide media by cyclic voltammetry and scanning electrochemical microscopy. *J. Am. Chem. Soc.* **136**, 311–320 (2014).

64. J. E. Frencken, P. Sharma, L. Stenhouse, D. Green, D. Laverty, T. Dietrich, Global epidemiology of dental caries and severe periodontitis - A comprehensive review. *J. Clin. Periodontol.* **44**, S94–S105 (2017).

65. M. Nazir, A. Al-Ansari, K. Al-Khalifa, M. Alhareky, B. Gaffar, K. Almas, Global prevalence of periodontal disease and lack of its surveillance. *Sci. World J.* **2020**, 1–8 (2020).

66. Global Health Metrics, "Caries of permanent teeth - Level 4 cause," 2019; www.healthdata.org/sites/default/files/disease_and_injury/gbd_2019/topic_pdf/cause/682.pdf.

67. Global Health Metrics, "Periodontal disease - Level 4 cause," 2019; www.thelancet.com/pb-assets/Lancet/gbd/summaries/diseases/periodontal-diseases.pdf.

68. N. Høiby, T. Bjarnsholt, M. Givskov, S. Molin, O. Ciofu, Antibiotic resistance of bacterial biofilms. *Int. J. Antimicrob. Agents* **35**, 322–332 (2010).

69. J. Kirsch, M. Hannig, P. Winkel, S. Basche, B. Leis, N. Pütz, A. Kensche, C. Hannig, Author correction: Influence of pure fluorides and stannous ions on the initial bacterial colonization in situ. *Sci. Rep.* **10**, 5695 (2020).

70. H. Deyu, Y. Li, L. Mateo, G. Xu, C. Myers, Y.-P. Zhang, N. Li, D. Rajah, Stannous fluoride vs regular fluoride dentifrice in plaque and gingivitis reduction. *J. Dent. Res.* **101**, 514 (2021).

71. S. Nathoo, B. G.-G. Socias, L. Mateo, C. Myers, G. Xu, M. Ryan, Y.-P. Zhang, Clinical efficacy on tooth sensitivity relief of SnF toothpaste. *J. Dent. Res.* **101**, 760 (2021).

72. S. N. Muddukrishna, A. Chen, T. R. Sykes, A. A. Noujaim, Indirect iodometric procedure for quantitation of Sn(II) in radiopharmaceutical kits. *Appl. Radiat. Isot.* **45**, 293–299 (1994).

73. X. Lu, T. Cheng, Y. V. Geletii, C. L. Hill, Catalytic system for aerobic oxidation that simultaneously functions as its own redox buffer. *Inorg. Chem.* **62**, 2404–2414 (2023).

74. B. Halliwell, M. Whiteman, Measuring reactive species and oxidative damage in vivo and in cell culture: How should you do it and what do the results mean? *Br. J. Pharmacol.* **142**, 231–255 (2004).

75. D. Darr, I. Fridovich, Free radicals in cutaneous biology. *J. Invest. Dermatol.* **102**, 671–675 (1994).

76. B. A. Jurkiewicz, G. R. Buettner, EPR detection of free radicals in UV-irradiated skin: Mouse versus human. *Photochem. Photobiol.* **64**, 918–922 (1996).

77. T. Herrling, J. Fuchs, J. Rehberg, N. Groth, UV-induced free radicals in the skin detected by ESR spectroscopy and imaging using nitroxides. *Free Radic. Biol. Med.* **35**, 59–67 (2003).

78. U. P. Strauss, T. L. Treitler, Degradation of polyphosphates in solution. I. Kinetics and mechanism of the hydrolysis at branching points in polyphosphate chains[1]. *J. Am. Chem. Soc.* **78**, 3553–3557 (1956).

79. U. P. Strauss, J. W. Day, Degradation of polyphosphates in solution. II. Hydrolysis of sodium hexaphosphate. *J. Polym. Sci. C Polym. Symp.* **16**, 2161–2169 (1967).

80. U. P. Strauss, G. J. Krol, Degradation of polyphosphates in solution. III. Hydrolysis of linear long-chain sodium polyphosphate. *J. Polym. Sci. C Polym. Symp.* **16**, 2171–2179 (2007).
